# Mitochondrial genome of the jack silverside, *Atherinopsis californiensis* (Atherinopsidae, Atheriniformes), a nearshore fish of the California Current Ecosystem

**DOI:** 10.1080/23802359.2022.2158047

**Published:** 2023-01-01

**Authors:** Janae E. Shew, Sean C. Lema

**Affiliations:** Biological Sciences Department, Center for Coastal Marine Sciences, California Polytechnic State University, San Luis Obispo, CA, USA

**Keywords:** Silverside, jacksmelt, mitochondrial DNA, genome, genetics

## Abstract

The jack silverside (*Atherinopsis californiensis*), also referred to as jacksmelt, is a neotropical silverside fish that inhabits nearshore shallow waters of the California Current Ecosystem in the Northeast Pacific Ocean, ranging from the coast of Oregon, USA, in the north to as far south as Baja California, Mexico. This fish is the sole member of its genus and is a commonly taken species when hook-and-line fishing in pelagic-neritic environments including bays, estuaries, kelp forests, and along sand beaches. Here we report the first complete mitochondrial genome of jack silverside consisting of 16,519 bp nucleotides and encoding 13 protein-coding regions, 12S and 16S rRNAs, 22 tRNAs, and an 841 bp D-loop control region. Phylogenetic analysis using all protein-coding genes of the complete mitogenome confirmed the inclusion of *A. californiensis* within subfamily Atherinopsinae of family Atherinopsidae, order Atheriniformes. This complete mitochondrial DNA genome will be of use for biodiversity assessments in the California Current ecosystem, while also providing a foundation for future mtDNA population genetic studies on this prominently caught species in shore- and pier-based recreational sport fishing.

## Introduction

The jack silverside (*Atherinopsis californiensis*, Girard, 1854) – also commonly referred to as ‘jacksmelt’ – is a pelagic schooling fish that reaches up to ∼20 cm in length and lives for 9–10 years (Clark 1929). *A. californiensis* ranges from Yaquina Bay, Oregon in the Pacific Ocean to as far south as Bahía Magdalena, Baja California Sur, Mexico (De La Cruz-Agüero et al. 1994). This species is generally considered to be omnivorous and consumes a broad range of food types (Barry et al. 1996; Horn et al. 2006; Higgins and Horn 2014). That versatility in diet allows both adult and juvenile *A. californiensis* to occupy a variety of open shallow water habitats, typically ranging in depths from approximately 1.5–15 m, from within and beyond the surf zone along sand beaches to estuaries and kelp forests (Horn 1979; Allen and Pondella 2006). With a nearshore distribution, *A. californiensis* is commonly caught by recreational sport anglers fishing from boats or shore (Ono 1982; Hill and Schneider 1999; Jarvis et al. 2004).

## Materials

**Figure 1.**
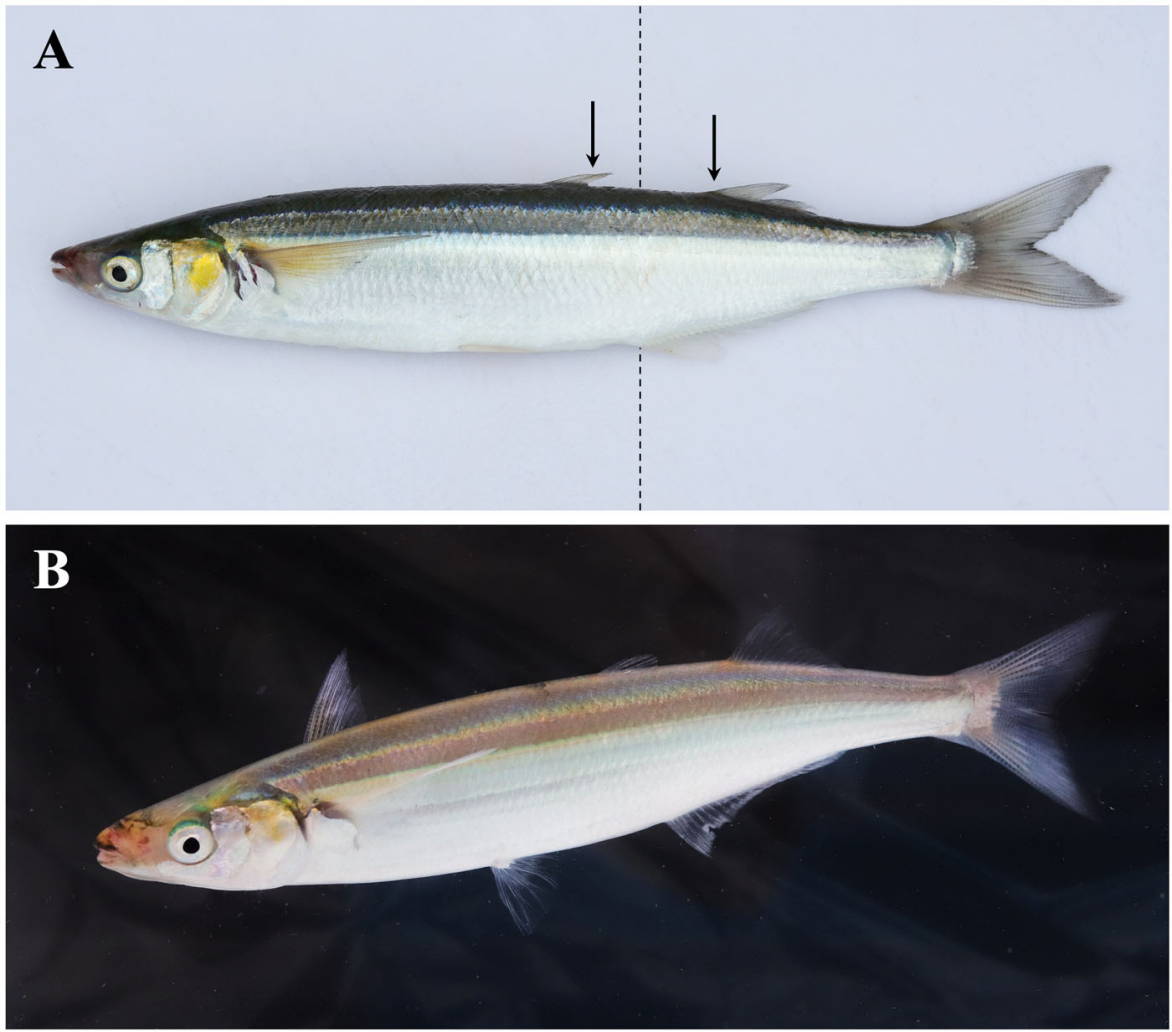
Photos of the jack silverside, *Atherinopsis californiensis*. (A) Jack silverside, commonly also called jacksmelt, belongs to the family of silversides (family Atherinopsidae) and not the taxon of ‘true’ smelts (family Osmeridae). Jack silverside are distinguished from another morphologically similar species – the topsmelt silverside (*Atherinops affinis*) – by the anterior edge of the anal fin (*dotted line*) being positioned between the posterior edge of the first dorsal fin and anterior boundary of the second dorsal fin (*arrows*). (B) Adult jack silverside showing the typical yellow color on the gill operculum, with positioning of the two dorsal fins stretched upright. Photos by Sean Lema.

Here we report the first complete mitochondrion DNA genome for *A. californiensis.* Skeletal muscle tissue was dissected from an adult jack silverside (Figure 1) (mass: 216.49 g; standard length: 25.8 cm; total length: 30.9 cm) collected on 21 September 2018 from Estero Bay near Cayucos, California, USA (35.445556 N, 120.908056 W). A sample of that tissue has been archived in the collections of the Natural History Museum of Los Angeles County, Los Angeles, California, USA (Dr. William Ludt, Assistant Curator, Ichthyology: wludt@nhm.org) under tissue no. LACM T-001462.

## Methods

DNA was extracted from the skeletal muscle using the DNeasy Blood and Tissue Kit (Qiagen, Valencia, CA), analyzed for quality and quantity both fluorometrically (Qubit 2.0 Fluorometer, Life Technologies, Carlsbad, CA) and by gel electrophoresis. DNA was then fragmented into a ∼350 bp library (Illumina Nextera DNA Flex Library Prep kit) and sequenced on the Illumina NovaSeq 6000 System (Novogene, Sacramento, CA) to generate ∼125 million 150 bp PE reads (GenBank BioSample no. SAMN27483993, Run Sequence Read Archive no. SRR18689888). Raw read quality was evaluated using FastQC version 0.11.9 (Andrews 2010). Raw 150 bp sequences were then assembled into a complete mitogenome using SMART2 (Alqahtani and Mandoiu 2020) using a partial sequence of the *A. californiensis* cytochrome b (*cytb*) gene (GenBank accession no. JQ282018; Bloom et al. 2012) as the ‘seed sequence’ for mitogenome assembly. SMART2 assembly generated a 16,519 bp nucleotide complete mitogenome from 400,000 of the raw 150PE sequences (median coverage depth: 26X). The complete mitogenome was subsequently confirmed in Galaxy version 21.09 (https://usegalaxy.org/) by going back to the raw 150 bp reads and conducting a separate alignment of those reads to the mitogenome assembled from the SMART2 pipeline. For that confirmational assembly, sequencing adaptors were first trimmed from the raw reads using fastp (Chen et al. 2018), and the trimmed sequences were aligned to the SMART2-generated mitogenome using Bowtie2 (Langmead and Salzberg 2012). Trimmed sequences that aligned to the SMART2 mitogenome were then downloaded and reassembled in Sequencher version 5.4.6 software (GeneCodes Corp., Ann Arbor, MI) for final confirmation of the complete mitogenome sequence. The resulting mitogenome was annotated using MitoFish (Iwasaki et al. 2013; Sato et al. 2018).

To confirm evolutionary relationships of *A. californiensis*, a maximum-likelihood phylogenetic tree was constructed using all amino acid coding sequence (CDS) regions concatenated from the mitogenomes of *A. californiensis* and eleven other fishes of Order Atheriniformes. Sequences were aligned using Clustal X software (Larkin et al. 2007), and the tree was constructed using all sites with pairwise gap deletion in MEGA version 11 (Tamura et al. 2021). Percent confidence values for each node were calculated from 1000 bootstrap replicates. The tree was rooted using the complete mitogenome from the Northern Anchovy, *Engraulis mordax* (MH613715) (Lewis and Lema 2019).

## Results

**Figure 2.**
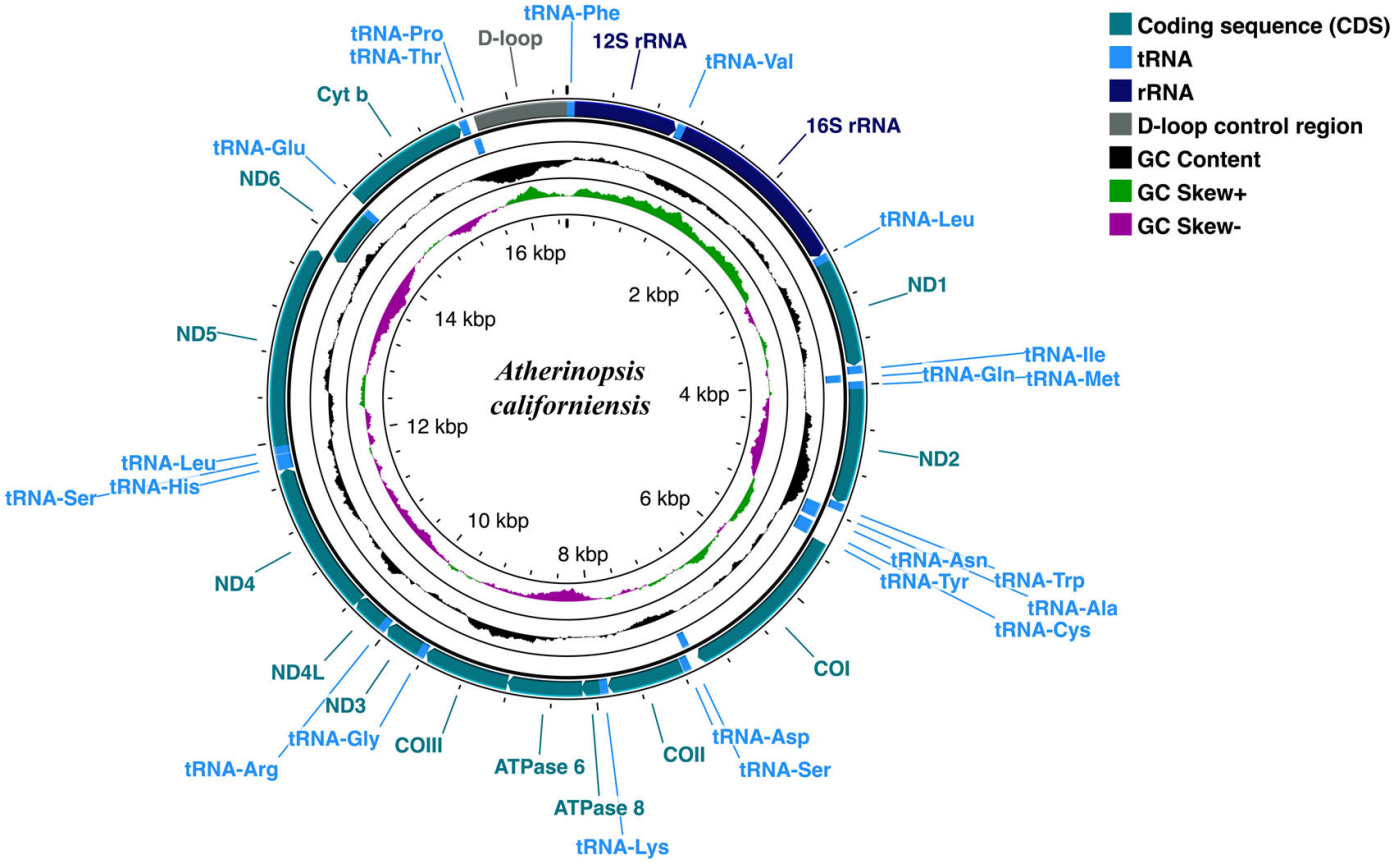
Circular sketch map of the complete mitogenome of the jack silverside *Atherinopsis californiensis*. Positions of amino acid coding sequence (CDS) genes, rRNAs, and tRNAs are indicated, as is GC nucleotide composition variation. Visualization generated using the Proksee server (https://proksee.ca/), which utilizes GCView (Stothard and Wishart 2005) for circular genome drawing.

The 16,519 bp complete mitogenome of jack silverside (GenBank accession no. ON310810) contains 13 protein-coding genes, 22 tRNAs, and 12S and 16 rRNAs in the typical arrangement of mitochondrial genes for actinopterygian fishes (Table 1). Of the 13 protein-coding genes, only *nd6* was encoded on the light strand (L-strand), and all others were found on the heavy strand (H-strand) (Figure 2). Similarly, eight of the tRNA genes (*tRNA^Gln^, tRNA^Ala^, tRNA^Asn^, tRNA^Cys^, tRNA^Tyr^, tRNA^Ser^, tRNA^Glu^,* and *tRNA^Pro^*) were located on the L-strand, while the other 14 tRNAs and both rRNAs were positioned on the H-strand. Overall nucleotide composition of the mitogenome had a GC content of 46.63% composed of the following: A, 26.24%; T, 27.13%; G, 17.34%; and C, 29.29%.

**Table 1.** Location and arrangement of genes on the mitogenome of *A. californiensis.*

Gene		Position^b^			Codon			
	Strand^a^	From (bp)	To (bp)	Size (bp)	Start	Stop^c^	# of amino acids	Intergenic spacer (bp)^d^
*tRNA^Phe^*	H	1	69	69				
*12S rRNA*	H	70	1012	943				
*tRNA^Val^*	H	1013	1085	73				
*16S rRNA*	H	1086	2779	1694				
*tRNA^Leu^*	H	2780	2853	74				
*ND1*	H	2854	3828	975	ATG	TAG	325	4
*tRNA^Ile^*	H	3833	3902	70				
*tRNA^Gln^*	L	(3902)	(3972)	71				
*tRNA^Met^*	H	3972	4040	69				
*ND2*	H	4041	5085	1045	ATG	T-	349	0
*tRNA^Trp^*	H	5086	5157	72				
*tRNA^Ala^*	L	(5159)	(5227)	69				
*tRNA^Asn^*	L	(5229)	(5301)	73				
*tRNA* ^*c*^ *^ys^*	L	(5340)	(5405)	66				
*tRNA^Tyr^*	L	(5406)	(5475)	70				
*COI*	H	5477	7027	1551	GTG	TAA	516	19
*tRNA^Ser^*	L	(7047)	(7117)	71				
*tRNA^Asp^*	H	7121	7192	72				
*COII*	H	7199	7889	691	ATG	T-	231	0
*tRNA^Lys^*	H	7890	7963	74				
*ATPase8*	H	7965	8132	168	ATG	TAA	56	−8
*ATPase6*	H	8123	8805	683	ATG	TA-	228	0
*COIII*	H	8806	9590	785	ATG	T-	262	0
*tRNA^Gly^*	H	9591	9661	71				
*ND3*	H	9662	10,010	349	ATG	T-	117	0
*tRNA^Arg^*	H	10,011	10,079	69				
*ND4L*	H	10,080	10,376	297	ATG	TAA	99	−5
*ND4*	H	10,370	11,750	1381	ATG	T-	461	0
*tRNA^His^*	H	11,751	11,819	69				
*tRNA^Ser^*	H	11,820	11,887	68				
*tRNA^Leu^*	H	11,892	11,964	73				
*ND5*	H	11,965	13,803	1839	ATG	TAA	613	−2
*ND6*	L	(13,800)	(14,321)	522	ATG	CAT	174	0
*tRNA^Glu^*	L	(14,322)	(14,389)	68				
*Cyt b*	H	14,394	15,534	1141	ATG	T-	381	0
*tRNA^Thr^*	H	15,535	15,608	74				
*tRNA^Pro^*	L	(15,608)	(15,677)	70				
*D-loop*	–	15,678	16,519	69				

^a^H and L indicate position on the heavy H or light (L) strand.

^b^Nucleotide (bp) positions in parentheses indicate encoded on complementary, light (L) strand.

^c^Codons containing ‘-’ symbols indicate an incomplete stop codon.

^d^Values correspond to the number of nucleotides separating this and the next gene or RNA. Negative numbers denote overlapping positions.

**Figure 3.**
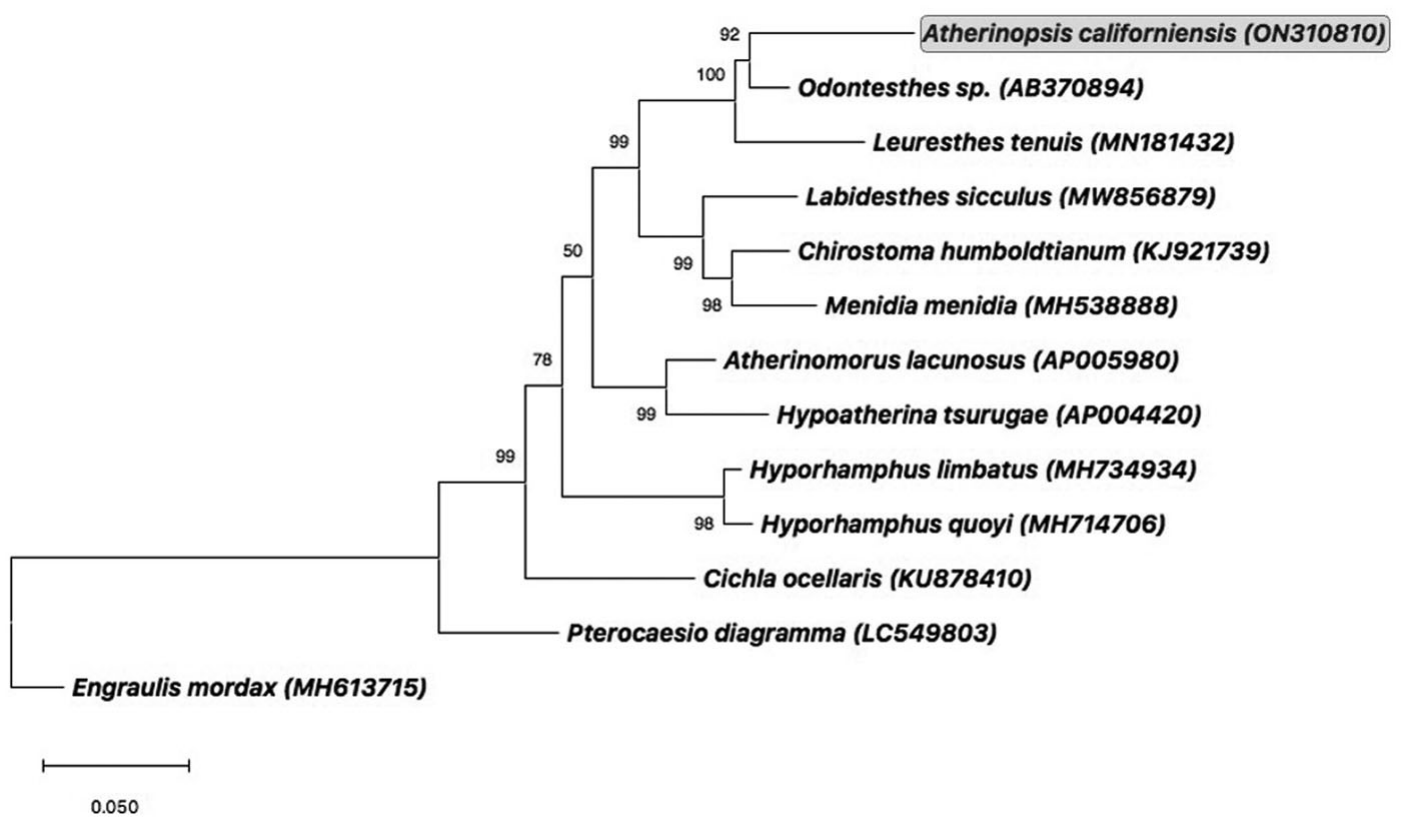
Consensus maximum-likelihood phylogenetic tree of the complete mitogenome of jack silverside *Atherinopsis californiensis* (GenBank accession no. ON310810; shown in *grey box*) and other fishes of Order Atheriniformes. GenBank accession numbers are provided in parentheses accompanying each taxon. The tree was rooted using the complete mitogenome from the Northern Anchovy, *Engraulis mordax* (Lewis and Lema 2019). Additional information on previously published complete mitogenomes for the following species used in this phylogenetic analysis can be found as follows: *Odontesthes* sp. (AB370894) (Setiamarga et al. 2008); *Leuresthes tenuis* (MN181432) (Muñoz et al. 2020); *Chirostoma humboldtianum* (KJ921739) (Barriga-Sosa et al. 2016); *Menidia menidia* (MH538888) (Lou et al. 2018); *Hypoatherina tsurugae* (AP004420) (Miya et al. 2003); *Hyporhamphus limbatus* (MH734934) (Lü et al. 2018); *Hyporhamphus quoyi* (MH714706) (Zhu et al. 2018), and *Cichla ocellaris* (KU878410) (Lin et al. 2017).

Phylogenetic analysis of protein-coding genes confirmed that *A. californiensis* belongs to the clade of Neotropical silverside fishes of subfamily Atherinopsinae, family Atherinopsidae (Figure 3). That phylogenetic arrangement corroborated the close relationship of *A. californiensis* to California grunion (*Leuresthes tenuis*) (Muñoz et al. 2020), which agrees with the relationship of *A. californiensis* to *Leuresthes* fishes observed previously using allozyme variation (Crabtree 1987).

## Discussion and conclusion

Although *A. californiensis* has received little research attention due to the species not being a target of commercial fisheries, *A. californiensis* is commonly caught in nearshore saltwater recreational sport fishing and is regularly consumed (Jarvis et al. 2004). The complete mitochondrial genome for *A. californiensis* reported here supports the evolutionary relationships of this species within subfamily Atherinopsinae, family Atherinopsidae, and provides a foundation for future assessments to fill a data gap on the population structure and genetic diversity of *A. californiensis* across its geographic range. Studies into how fishing and climate change are affecting marine fish biodiversity in the California Current Ecosystem are increasingly utilizing environmental DNA (eDNA) and other high throughput DNA sequencing approaches (e.g. Closek et al. 2019; Stat et al. 2019). The cytochrome oxidase I (*coi*) sequence from this new mitogenome and other sequences from *A. californiensis* (e.g. KM019227-KM019229) show >7% nucleotide sequence divergence compared to topsmelt silverside *Atherinops affinis* (OL806589-OL806590), the closest relative of *A. californiensis*, and ∼10% divergence with California grunion (*L. tenuis*, MN181432), indicating *coi* is a viable target for distinguishing these species in large-scale, DNA-based studies in the California Current Ecosystem.

## Data Availability

The mitochondrion genome sequence data that support the findings of this study are openly available in GenBank of the National Center for Biotechnology Information (NCBI) at https://www.ncbi.nlm.nih.gov/ under accession no. ON310810. Raw 150PE reads generated by the authors to assemble this mitogenome are available under GenBank BioProject no. PRJNA824876, BioSample no. SAMN27483993, and Run Sequence Read Archive no. SRR18689888.
